# Tracheobronchial Amyloidosis Mimicking Tracheal Tumor

**DOI:** 10.1155/2016/1084063

**Published:** 2016-08-09

**Authors:** Elif Tanrıverdi, Mehmet Akif Özgül, Oğuz Uzun, Şule Gül, Mustafa Çörtük, Zehra Yaşar, Murat Acat, Naciye Arda, Erdoğan Çetinkaya

**Affiliations:** ^1^Department of Chest Diseases, Yedikule Chest Diseases and Thoracic Surgery Training and Research Hospital, Kazlıçeşme, Belgrat Kapı yolu Cad. 1, Zeytinburnu, 34020 Istanbul, Turkey; ^2^Department of Chest Diseases, Ondokuz Mayıs University Faculty of Medicine, Samsun, Turkey; ^3^Department of Chest Diseases, Karabük University Faculty of Medicine, Karabük, Turkey; ^4^Department of Chest Diseases, Abant İzzet Baysal University Faculty of Medicine, Bolu, Turkey; ^5^Department of Pathology Yedikule Chest Diseases and Thoracic Surgery Training and Research Hospital, Kazlıçeşme, Belgrat Kapı yolu Cad. 1, Zeytinburnu, 34020 Istanbul, Turkey

## Abstract

Tracheobronchial amyloidosis is a rare presentation and accounts for about 1% of benign tumors in this area. The diagnosis of disease is delayed due to nonspecific pulmonary symptoms. Therapeutic approaches are required to control progressive pulmonary symptoms for most of the patients. Herein, we report a case of a 68-year-old man admitted with progressive dyspnea to our institution for further evaluation and management. He was initially diagnosed with and underwent management for bronchial asthma for two years but had persistent symptoms despite optimal medical therapy. Pulmonary computed tomography scan revealed severe endotracheal stenosis. Bronchoscopy was performed and showed endotracheal mass obstructing 70% of the distal trachea and mimicking a neoplastic lesion. The mass was successfully resected by mechanical resection, argon plasma coagulation (APC), and Nd-YAG laser during rigid bronchoscopy. Biopsy materials showed deposits of amorphous material by hematoxylin and eosin staining and these deposits were selectively stained with Congo Red. Although this is a rare clinical condition, this case indicated that carrying out a bronchoscopy in any patient complaining of atypical bronchial symptoms or with uncontrolled asthma is very important.

## 1. Introduction

Amyloidosis is characterized by the abnormal extracellular deposition of insoluble fibril proteins. Diseases are detected as systemic amyloidosis or localized amyloidosis. Systemic amyloidosis is classified into four categories as primary, immunoglobulin light chain (AL) disease; secondary, amyloid A protein (AA) disease; hereditary, mutant transthyretin (ATTR) disease; dialysis-dependent, beta 2 microglobulin (*β*2m) disease. Localized amyloidosis is more common than systemic amyloidosis. Localized amyloidosis is limited single organ such as bladder, skin, heart, or lung [1, 2]. The most cases of respiratory amyloidosis have been described in three forms: diffuse interstitial amyloidosis, nodular parenchymal amyloidosis, and tracheobronchial amyloidosis [3]. With only around one hundred cases reported in the literature, tracheobronchial amyloidosis is a rare form of localized amyloidosis [4].

## 2. Case Reports

68-year-old male patient was admitted to our outpatient clinic with complaints of dyspnea and cough. He was followed with a diagnosis of bronchial asthma for two years. But despite his adherence to the treatment, his symptoms worsened with a progressive decrease in exercise tolerance, increased wheezing episodes, and shortness of breath. Chest tomography (CT) was performed for ruling out obstructive lesions. CT demonstrated tracheal tumor which obstructed tracheal lumen. His medical history revealed appendectomy operation and two times of hernia operations. Also, he had the diagnosis of diabetes mellitus (DM), hypertension (HT), benign prostatic hypertrophy (BPH), and arrhythmia. He was taking treatment for these diseases.

**Figure 1:**
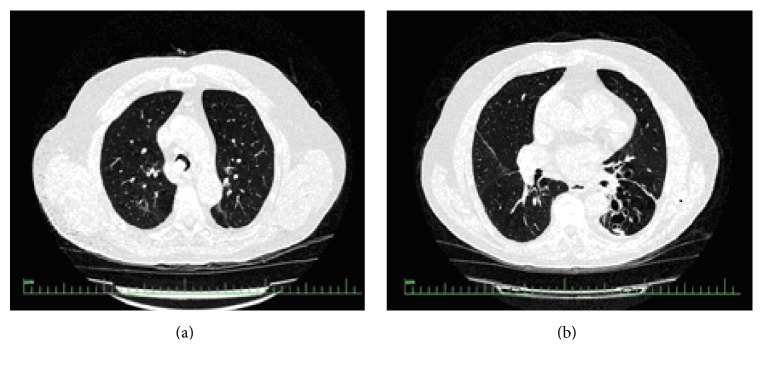
Computed tomography scan revealed the mass in the lumen of trachea (a) and cystic bronchiectasis on left lower lobe (b).

**Figure 2:**
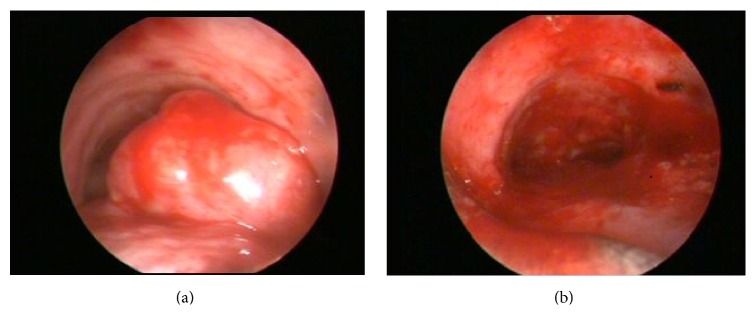
Endoscopic visualization shows the mass protruding from the posterior wall of distal trachea (a) and after complete resection of the mass (b).

**Figure 3:**
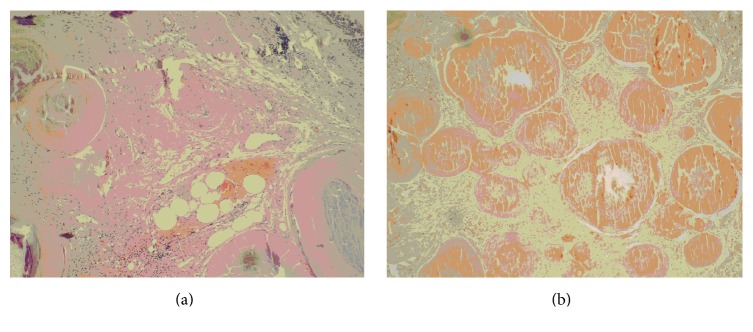
The calcification in tissue and the deposition of amorphous material (HE ×200) (a) and deposits of amyloid materials stained with Congo Red (Congo Red ×200) (b).

On physical examination, blood pressure was 140/90 mmHg, respiratory rate was 17/min, and heart rate was 80/min and arrhythmic. There was an inspiratory rale on the left hemithorax on pulmonary auscultation. Routine laboratory test results were within normal limits. Forced expiratory volume in 1 s (FEV1) was 1.54 L (70.4% of predicted value), forced vital capacity (FVC) was 2.07 L (73.9% of predicted value), and FEV1/FVC ratio was 0.68 on his spirometry. He had bronchodilator response as 12.57% increase in the FEV1. Chest tomography (CT) scan revealed the mass in the lumen of trachea and cystic bronchiectasis on left lower lobe (Figure 1). Bronchoscopy was performed and showed a mass obstructing 70% of the distal trachea and spreading to right main bronchus (Figure 2). The patient underwent rigid bronchoscopy and the mass was removed by mechanical resection following both Nd-YAG laser and argon plasma coagulation. Initially, we preferred Nd-YAG laser for mass lesion resection purpose. However, we encountered a breakdown on Nd-YAG laser device. Therefore, we kept on the procedure with argon plasma coagulation. Cryotherapy was performed for residual tissue. After the removal of the debris, tracheal lumen patency were obtained (Figure 2). The procedure was well tolerated and the respiratory symptoms disappeared rapidly. In the histopathological examination of the material, hematoxylin and eosin staining showed the presence of calcified eosinophilic amorphous materials (Figure 3) which was selectively stained with Congo Red (Figure 3). Histopathological evaluation revealed that the tumor was tracheobronchial amyloidosis. We did not use adjuvant treatment in this case and he is alive with no evidence of recurrence after 25 months from the first bronchoscopy. Bronchoscopy was performed at 12 months later in another medical center, reported to be normal. Recently, we advised performing a bronchoscopy but the patient did not accept since he feels ok.

## 3. Discussion

Tracheobronchial amyloidosis (TBA) is an uncommon localized form of amyloidosis, characterized by abnormal amyloid deposits in the trachea and main bronchi [5]. Amyloidosis commonly affects males (2 : 1) in middle age (50–60 years) groups [6]. Our case was male as in literature but was not 50–60 years old because of delayed diagnosis. The clinical presentations of the TBA vary depending on the involved area. In the case of pulmonary involvement, the patients sometimes have no symptoms. But common presenting symptoms include cough (74%), wheezing (70%), dyspnea (60%), hemoptysis (50%), and stridor (30%). Our patient firstly suffered from cough and sputum; after that, progressive dyspnea added to his complaints.

One group of these patients have normal chest X-ray but other groups of them (>50%) might have obstructive pneumonia, bronchiectasis, or atelectasis. This condition requires differential diagnosis of various diseases such as asthma, recurrent pneumonia, and malignancy [6]. Our patient was followed up and threated like asthma for two years. But his pulmonary symptoms were progressive in spite of therapy.

Thorax CT is so sensitive for detection of changes associated with amyloidosis. Submucosal wall thickening with irregular calcifications and luminal narrowing can be seen in the trachea and main bronchi [7]. These conditions are seen like diffuse submucosal plaques on bronchoscopic examination. Only 14% of the cases have solitary deposits and these deposits mimic endobronchial malignancy [8]. The initial form of presentation in this case is unusual. The amyloidosis manifested as an isolated mass, which suggested malignant disease, causing almost complete occlusion of the distal part of the trachea.

TBA in the central airways is not suitable for surgery. Endobronchial therapy is the only treatment that allows airway patency [9]. Fiorelli et al. reported their case performing Y self-expanding stent implantation after endobronchial laser resection. They showed significant clinical success with this case [9]. Laser photocoagulation is good endobronchial treatment for amyloidosis because of amyloid's sensitivity. But this treatment should be performed for localized lesions [7]. We performed APC and laser photocoagulation to our case due to localized tumor. There were no complications during endobronchial therapy.

Tracheobronchial amyloidosis must be included among the differential diagnoses of tracheal lesions such as tracheal neoplasms, tuberculosis, inflammatory fibroepithelial polyp, and papillomatosis. Therefore, bronchoscopy and tissue biopsy are essential for the diagnosis of tracheobronchial amyloidosis. The diagnosis of amyloidosis usually requires histological confirmation. Tissue samples taken from endobronchial lesions included homogeneous lamellar material accumulation and this material was stained with Congo Red. These findings are specific and gold standard for amyloidosis.

Although TBA is localized disease, two series reported low survival rate like 31% (13 of 41 patients) and 43% (3 of 7 patients) at 4–6 years of age. Other case series with mid airway diseases demonstrated that 7 of 15 patients (47%) progressed to respiratory failure. According to these reports, the average life span of these patients was only about 9 years after diagnosis. Morbidity and mortality were associated with quantity and rate of amyloid deposition in the airways [10].

In our case, endobronchial lesion covered a large area of tracheal lumen as malignant tumor. His symptoms were similar to asthma and he was treated for 3 years. But he had significant clinical improvement after endobronchial therapy. We thought that all clinicians should be alert particularly in patients not responding to treatment.

Consequently, TBA is rare disease that can be diagnosed by histopathology. Early diagnosis is important to prevent complications. Treatment is usually by endobronchial methods. We thought that endobronchial therapy can be safely performed in patients with TBA as in our case.
